# A scoping review on pathways/models/frameworks for palliative care provision for cancer patients while admitted at hospitals within sub-Saharan Africa

**DOI:** 10.3332/ecancer.2026.2104

**Published:** 2026-03-31

**Authors:** Agnes Chipo Tererai, Margaret Borok, Zvavahera Mike Chirenje, Liz Gwyther, Lindsay Farrant, Ntokozo Ndlovu, Simbarashe Rusakaniko

**Affiliations:** 1 Department of Oncology, Medical Physics and Imaging Sciences, University of Zimbabwe, PO Box A178 Avondale, Harare, Zimbabwe; 2 Department of Medicine, College of Health Sciences, University of Zimbabwe, PO Box A178 Avondale, Harare, Zimbabwe; 3 Clinical Trials Research Centre, University of Zimbabwe, PO Box A178 Avondale, Harare, Zimbabwe; 4 Department of Obstetrics, Gynaecology and Reproductive Science, University of California San Francisco, San Francisco, CA, USA; 5 Department of Family, Community and Emergency Care, University of Cape Town, E47-25, Observatory, Care, Cape Town 7925, South Africa; 6 Department of Community Medicine, College of Health Sciences, University of Zimbabwe, PO Box A178 Avondale, Harare, Zimbabwe; a https://orcid.org/0009-0006-3822-9582; b https://orcid.org/0000-0002-1234-0913; c https://orcid.org/0000-0003-3538-5150; d https://orcid.org/0000-0001-7602-6168; e https://orcid.org/0000-0002-1997-429X; f https://orcid.org/0000-0002-8269-7571; g https://orcid.org/0000-0002-3159-0205

**Keywords:** cancer, care pathway(s)/models/framework, hospital(s), palliative care, Sub-Sahara Africa

## Abstract

**Background:**

Tertiary hospitals are multi-specialty centers where patients are referred for various treatment modalities, including cancer diagnosis and treatment. Often, cancer patients are admitted to hospitals several times. Palliative care (PC) promotes quality of life for patients, their families and caregivers and should be integrated into cancer care at hospitals.

**Aim:**

To review literature on PC pathways, models and frameworks for cancer care in hospitals.

**Setting:**

Hospitals in sub-Saharan Africa (SSA).

**Methods:**

The scoping review was conducted following the Preferred Reporting Items for Reviews and Meta-Analysis extension for Scoping Reviews checklist. Four databases (Medline, Embase, AMED and Scopus) were searched. Inclusion criteria were PC, pathway/ model/framework, cancer, hospital, SSA countries and articles with abstracts. Each database was searched using OR to link synonymous words, then AND to combine all keywords. Two documents of grey literature related to the topic were included. Findings were tabulated under author, year, title, location, PC pathways and cancers. Analysis was done using descriptive methods.

**Results:**

Of the 264 articles screened, 15 (13 studies and two reports) were included. These were from eight SSA countries. Six of the studies had a model of PC provision in hospitals within the outpatient department or oncology unit and provided by any health worker and/or a trained PC team. The other studies did not have a model but highlighted the need for one.

**Conclusion:**

The need for PC for cancer patients in hospitals is recognised across SSA; however, countries are at different stages of developing hospital PC pathways.

**Contribution:**

Findings will guide the formulation of a PC pathway for cervical and prostate cancer patients at a tertiary hospital in Harare Zimbabwe.

## Introduction

### Background and rationale

It is important to understand the various palliative care (PC) pathways that can be used in the management of cancer within a hospital context. PC is recognised as a basic human right, especially for patients with serious health-related suffering. PC was first developed for patients with advanced cancer and for end-of-life care [1]. The escalating burden of cancer with the associated suffering, is global. The number of new cancer cases is projected to increase by more than 200% over the next 40 years in the low- to middle-income countries (LMICs), while the same figure is projected to be a 40% increase in high-income countries (HICs), due to an ever-increasing population experiencing declining, curable or treatable (non-recurrent) diseases [2]. Evidence indicates that when cancer care includes palliative and supportive care, it provides a better quality of life for patients, families and caregivers [3]. However, determining when and how to include supportive and PC within cancer management can be complex and requires an evidence-based approach to determine best practices [4]. There are different models/frameworks for integrating PC into cancer management as outlined by Hui *et al* [4], Bruera and Hui [5], Hui and Bruera [6, 7]. Kaasa *et al* [3] recommend the use of standard care pathways for the integration of oncology and PC. The Lancet commission on cancer in SSA recommended the five S framework (staff, stuff/services, space, systems, synergies) for improving access to PC in oncology [8].

PC integration into oncology helps to alleviate the suffering of cancer patients, their families and caregivers. It is important to have evidence from LMICs, especially from sub-Saharan Africa (SSA). Understanding the different PC pathways/models/frameworks used in cancer management at hospitals, especially within SSA could inform the establishment of a PC pathway at a tertiary hospital in Harare. According to the Cambridge dictionary a pathway is ‘a series of actions that can be taken to achieve something [9]’ and from Collins dictionary it is ‘a particular course of action or a way of achieving something [10]’. For this study, a pathway is described as a structured roadmap that guides a patient’s care journey with a life-limiting/threatening illness, specifically cancer in this study, ensuring their physical, psychosocial and spiritual needs are met by a multidisciplinary team. Therefore, literature on PC pathways as well as models and frameworks integrated into cancer management at hospitals, is explored in this scoping review. Literature describing models and frameworks contained elements that related to describing pathways.

A scoping review has been chosen over other review methods, such as systematic reviews because the researcher realises that there is limited information regarding PC pathways used in oncology at hospitals, especially in SSA context. The information from the scoping review will provide evidence for recommendations to policy makers and other important stakeholders in Zimbabwe to develop a suitable PC pathway for tertiary hospitals to address the increasing cancer burden.

#### Scoping review question

1. What are the described pathways/models/frameworks to PC provision for patients with cancer admitted at hospital in SSA?

#### Aim

To describe pathways/models/frameworks of PC provision within SSA for adult patients with cancer while admitted at hospital.

#### Objectives

1. To explore the pathway/model/framework to initiation of PC for cancer patients at hospitals in SSA using a scoping review.
2. To describe the subsequent PC pathways/model/framework for cancer patients in hospitals in SSA through a scoping review.

## Methods

The Preferred Reporting Items for Systematic reviews and Meta-Analysis extension for Scoping Reviews checklist [11] was used to guide the scoping review manuscript. A scoping review is useful to come up with literature on emerging topics and to identify any gaps [12]. This was done following the six steps as described by Mak and Thomas [13]:

Step 1: Identifying the research question.

Step 2: Identifying relevant studies

Step 3: Selecting studies to be included in the review

Step 4: Charting the data

Step 5: Collating, summarising and reporting the results

Step 6: Consulting stakeholders (optional)

The scoping review was conducted using population, concept and context (PCC) elements [14], derived from the research question: *what are the described pathways/models/frameworks to PC provision for patients with cancer while admitted at hospitals in SSA?* P = cancer patients, C = palliative care pathways and C = hospitals in SSA). Two articles of grey literature (Zimbabwe national PC policy and document on PC in women’s cancer care: Global challenges and advances) were purposively chosen as they contribute significantly to the information required for the formulation of a PC pathway for a tertiary hospital in Zimbabwe, which is the aim of the subsequent study. The use of the two grey literature articles was necessary to make the scoping review more complete since the database search for pathways in SSA did not provide the evidence. The 2018 FIGO report was included as grey literature since it emerged from some of the database searches and provided important information regarding PC within SSA. The other grey literature that was included is the Zimbabwe national PC policy and this was necessary to provide a closer look on PC within SSA by using an important document in PC integration efforts, which will feed into the main study in the Zimbabwean setup.

### Eligibility criteria

#### Inclusion criteria

1. Studies of adult cancer patients and/or cancer clinicians within SSA
2. Studies mentioning and including a PC pathway/framework/model for cancer(s),
3. Studies of cancer patients admitted in hospital (district, regional/provincial, tertiary)
4. Studies with abstracts,
5. Published studies,
6. Studies in English language or translated to English language
7. Studies from 1950 to 2024

#### Exclusion criteria

1. Studies of non-cancer patients or child cancer patients or cancer clinicians outside SSA.
2. Studies not mentioning and not including a PC pathway/framework/model for cancer.
3. Studies of cancer patients not admitted in hospital (e.g., long-term care facilities such as care homes, rehabilitation facilities or at home).
4. Studies without abstracts.
5. Unpublished studies.
6. Studies in languages not translated to English.

### Information sources and search strategy

The main researcher (ACT) developed the search strategy with assistance from the librarian at the UZ Medical School library. Another independent researcher, LF, reviewed the search terms before ACT conducted the database searches.

Four electronic databases were searched:

1. Medline via PubMed
2. Embase via OVID
3. AMED via OVID
4. Scopus via OVID

The databases were selected with the librarian’s help to ensure coverage of the required information and to complement each other by providing articles from various health disciplines related to PC. The search period was from 1950 to December 2025. Each database was searched by combining keywords using OR for synonyms, then AND to link all terms together. Additional information was obtained from specific grey literature, including the Zimbabwe National PC Policy and the National Cancer Prevention and Control Strategy. The electronic referencing system used was ENDNOTE 21 (Clarivate, Philadelphia, PA, USA; London, UK).

Search terms: cancer, care pathway(s), hospital(s), PC, Sub-Sahara Africa.

The following terms were applied for each database search:

1. Cancer OR oncology OR malignancy AND
2. Care pathway OR Framework OR Model AND
3. Hospital OR health facility AND
4. PC OR end of life care OR hospice care OR supportive care AND
5. Sub-Sahara Africa (SSA) OR Africa south of the Sahara

Methodology for the literature search was advised by the librarian and to ensure SSA coverage. The librarian assisted in identifying the countries under SSA and how to undertake the literature search. MeSH and non-MESH terms that were synonymous with the search word were linked in the search with OR, then after independent searches were done AND was used to link the searches.

An additional source of information was obtained from a specific grey literature: the Zimbabwe national PC policy, while the second grey literature: PC in women’s cancer care: Global challenges and advances – 2018 FIGO report was obtained from the database searches.

A reference list of all the articles/papers in the final list is provided in Table 1. Citation followed the Vancouver referencing format. The electronic referencing system was ENDNOTE 21 (Clarivate, Philadelphia, PA, USA, London, UK.

Example of a search strategy from one database - Medline via Pubmed:

Search: **((((((cancer) OR (oncology)) OR (malignancy)) AND ((Sub-Sahara Africa) OR (Angola OR Benin OR Botswana OR Burkina Faso OR Burundi OR Cameroon OR Cape Verde OR Central African Republic OR Chad OR Comoros OR Dem. Rep. of the Congo OR Djibouti OR Equatorial Guinea OR Eritrea OR Ethiopia OR Gabon OR Gambia OR Ghana OR Guinea OR Guinea-Bissau OR Ivory Coast OR Kenya OR Lesotho OR Liberia OR Madagascar OR Malawi OR Mali OR Mauritania OR Mauritius OR Mayotte OR Mozambique OR Namibia OR Niger OR Nigeria OR Republic of the Congo OR Reunion OR Rwanda OR Sao Tome and Principe OR Senegal OR Seychelles OR Sierra Leone OR Somalia OR South Africa OR South Sudan OR St. Helena OR Swaziland OR Tanzania OR Togo OR Uganda OR Zambia OR Zimbabwe))) AND (((care pathway) OR (framework)) OR (model))) AND ((hospital) OR (health facility))) AND ((((palliative care) OR (end of life care)) OR (hospice care)) OR (supportive care))** Filters: **MEDLINE, from 1999 - 2024** Sort by: **Publication Date**

### Study selection/screening

Eligibility criteria, as previously stated, were used to screen studies, although these criteria were modified during review and piloting stages conducted by ACT and LF. ACT performed database searches, and references were exported to EndNote, then transferred to Rayyan, a web application used to manage systematic reviews for title and abstract screening [15].

After the pilot, ACT and LF independently screened the first 100 articles’ titles and abstracts using Rayyan, with blinding on, following the inclusion criteria. Subsequently, with blinding off, they compared their screening decisions for consistency. They met virtually five times over two months to discuss progress and troubleshoot challenges.

ACT conducted the full-text screening manually with guidance from LF. LG, a senior researcher and PC expert, reviewed the work prior to final draft production. ACT carried out the final full-text screening. A record of all studies from the beginning was maintained. The PRISMA flow chart below illustrates the selection process to reach the final eligible studies.

### Data collection, extraction and charting

ACT developed the data charting form using Excel spreadsheet template from Joanna Briggs Institute and sought advice from co-authors to agree the final form. The data charting form specified all the variables/elements to be collected: title, authors, publication journal, country, study design, study aim, study population, study setting (context), concept (PC pathway/framework/model) and notes. All included articles were read for data extraction by ACT.

### Synthesis and presentation of results

**Figure 1.**
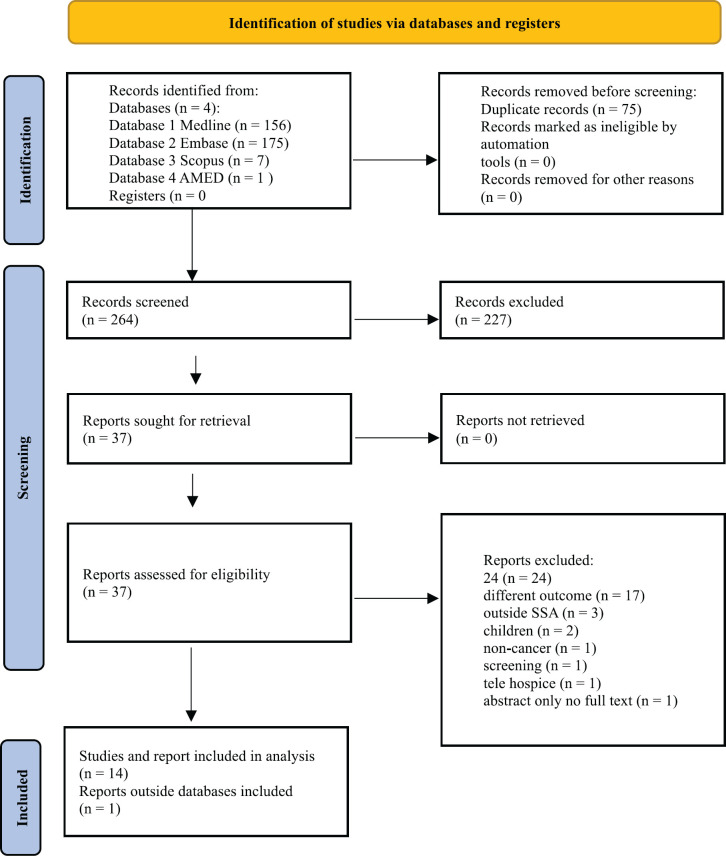
Scoping review PRISMA flow-chart [16].

A PRISMA flow diagram Figure 1 below shows the flow of initial record of articles total 264 from the four databases (Medline, Embase, Scopus and AMED) and progressive refinement through screening to the final list. Data cleaning was enhanced through the use of Microsoft Excel (Microsoft Corporation, Redmond, WA, USA). Data extraction tables and other forms were used to present the data. Descriptive narrative synthesis was done.

### Results and analysis of sources of evidence

Table 1 represents the operational matrix for the scoping review through which the articles were analysed. As described before, we included models and frameworks since there were no articles on pathways.

### Results and analysis of sources of evidence

Of the four databases used, Embase had the most articles 175, followed by Medline 156, then Scopus seven and lastly AMED had one. Embase and Medline had many similar studies, hence the duplicates at first screening were 75.

There were 15 final articles chosen, of which two were reports (one FIGO report and one National PC policy for Zimbabwe). The FIGO report by Cain and Denny in 2018 focused on PC in women’s cancer care and proposed a model that includes adequate human resources/training, equipment/services, policies and monitoring, as well as public awareness and education [22]. The Zimbabwe national PC policy states that:

6Sustainable PC is achieved through creating a favorable environment where the goals and service of PC can be achieved; leadership with an understanding of and experience in PC; strong coordination and collaboration; and legislation that supports an enabling environment for PC, that includes access to necessary medicines and human resources [31].

Six out of the 15 articles m*et al*l the three aspects of PCC with population being patients, clinicians, caregivers or stakeholders while the context was tertiary hospital or just reported as hospital and the concept mentioned by all the six was a model of PC [18, 20–23, 25]. There was no mention of a PC pathway for cancer patients at SSA hospitals in all 13 articles without including the two grey literature.

Three out of the six studies described hospitals with a PC clinic/unit, and this was reflected in the Zimbabwe PC policy. Seven of the studies did not mention model/framework but described the population and context and the context of PC provision was a PC unit [17], PC team for the hospital [19] and PC confined to oncology ward/cancer unit [26, 30].

The scoping review findings are summarised into articles which described high PC integration and defined models, moderate integration with partial elements and low integration with no model identified as shown in Figure 3 below.

A detailed discussion of the scoping review findings and their implications in the provision of PC within SSA follows below.

## Discussion

PC utilisation across SSA is influenced by multiple factors, including perceptions of PC, health-system limitations and infrastructural constraints. The study by Afessa *et al* [17] in Addis Ababa demonstrated that PC service utilisation among cancer patients is shaped by a combination of predisposing, enabling, health-system and need-related factors. However, despite recognition of the need for PC, utilisation remains minimal and this echoes findings from the SSA studies. Benefits of PC integration are clear, providing improved outcomes for patients, families and health systems, but effective model implementation is needed.

A consistent theme across the included studies is the absence of clearly defined or structured models for PC integration within hospital systems. Bonsu and Ncama (2019) highlight the need to develop explicit models and protocols to support the integration of breast cancer prevention and early detection into PC [18, 21]. Clinicians report limited guidance on care pathways and note that late presentations reduce survival prospects, underscoring the urgency for structured PC integration. PC integration into cancer within SSA is still very disjointed and there are not many studies on PC in this region. There seems to be a lack of integration across the oncology-PC continuum. This provides some explanation why there was no study in this scoping review that mentioned PC pathways and only six studies out of the 13 studies describing some form of PC model. This finding highlights the need for escalating strategies to increase PC integration, especially within LMICs where the cancer burden is increasing very fast compared to HICs.

Evidence from South Africa [19, 23] emphasises the importance of health-care worker training, strengthened communication skills and multidisciplinary support systems. Ganca *et al* [19] found that clinicians supported by hospital-based PC teams experience reduced emotional burden when delivering poor prognoses suggesting that integration of PC into hospital systems improves both clinician well-being and patient-family communication. This highlights the role of the multidisciplinary team in PC. Similarly, Hamilton-Baillie *et al* [23] show that sustained training and awareness initiatives can strengthen service provision even in rural settings. This scoping review showed PC provision in hospitals for cancer patients as unidirectional, bidirectional, as well as strong NGO engagement. However, because these were isolated and very few studies focused on few identified areas of countries, there is need for further studies within the SSA context to ascertain the best approach bearing in mind the unique needs of each country. The need for some guiding models in the care of cancer patients when PC provision is required was highlighted by Bonsu and Ncama and this is especially crucial in SSA where majority present late.

From a systems perspective, both the availability and organisational structure of PC services are essential. Ogbenna *et al* [20] conducted an environmental scan in Nigeria and identified variations in patient outcomes across five tertiary hospitals, linked to differences in PC unit leadership and organisational capacity. This variation underscores the need for scalable, adaptable PC models rooted in local contexts. Similarly, the Waterloo Coalition Initiative in Malawi [25] demonstrates that integration is possible even in resource-constrained environments when guided by structured indicators and committed partnerships.

Perception and awareness also influence PC access. Studies from Ethiopia [17, 30] show that patient awareness of PC and health worker attitudes significantly impact service uptake [17, 30]. Meanwhile, Zimbabwe’s policy framework (Ministry of Health and Child Care, 2014 [31] and Tapera and Nyakabau [29]) reveal that community-level services, such as Island Hospice, fill critical gaps left by under-resourced public facilities.

Barriers to PC integration identified by Ndiok and Ncama [27, 28] include inadequate policies, lack of training and limited funding, same challenges highlighted in the Botswana study by LaVigne *et al* [26]. Benefits of PC integration are clear; providing improved outcomes for patients, their families and health systems, but there is need for effective implementation of the models. In conclusion, while the value of PC need is growing in cancer care within SSA, PC service utilisation and integration remain hindered by systemic, structural and perceptual barriers. Key in addressing these barriers is having context-specific models for hospital PC provision, health-worker support and strong policies.

**Figure 2.**
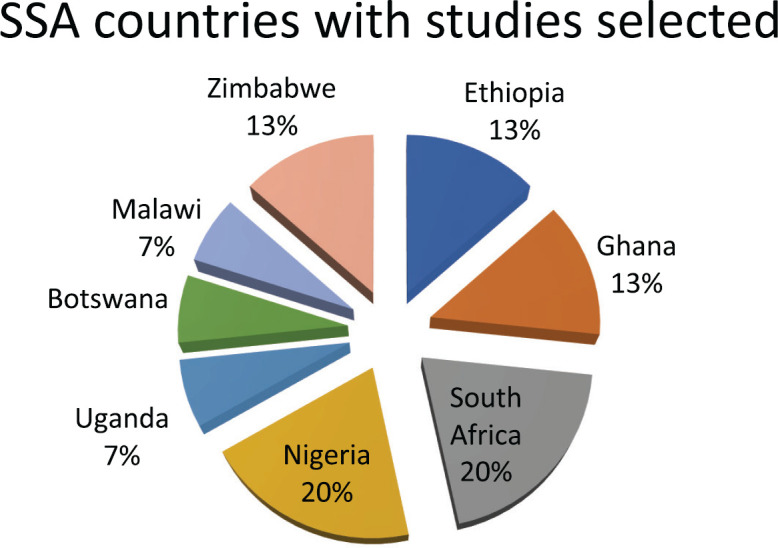
Countries with studies selected.

**Figure 3.**
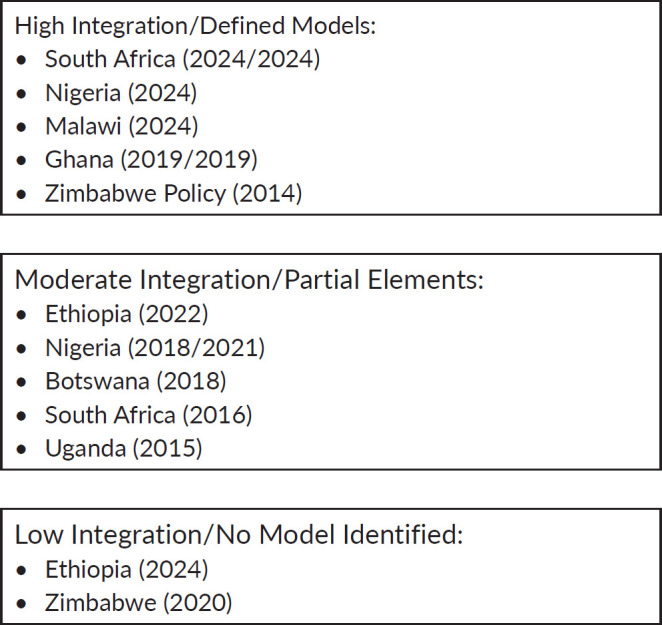
Continuum of PC Integration across included studies.

In conclusion, despite an increasing acknowledgment of the need for PC in cancer treatment throughout SSA, there are still many obstacles that hamper the implementation and usage of PC, including systemic, structural and societal-related issues. Strengthening PC provision requires context-specific models for hospital-based care, improved clinician support systems and stronger policy frameworks. Notably, although SSA comprises 49 countries, only studies from eight (16.3%) met the inclusion criteria for this review (Figure 2), highlighting a significant evidence gap. This gap mirrors the broader challenge of limited PC access despite a high and rising cancer burden in the region [32]. There is a high need for PC but low provision for access to PC in SSA. A study to come up with a clearly developed hospital pathway for PC integration into cancer management will be valuable in providing evidence for policy makers to integrate PC in SSA hospitals.

Developing a clearly defined hospital-based PC pathway could therefore provide critical guidance for policymakers and clinicians working to integrate PC into tertiary-level cancer care in Zimbabwe and beyond.

### Ethical considerations

The scoping review was conducted as the first part of the main study, whose ethical approval has been obtained from the review committee of Parirenyatwa-University of Zimbabwe joint research ethics committee (JREC ref 394/2025) and the Medical Research Council of Zimbabwe (MRCZ/A/3368). The scoping review did not require human subjects but made use of results from other studies conducted before, hence there was no need for ethical approvals to conduct it.

### Dissemination

The findings will be shared with the UZ, GHAP team and other PC and oncology groups at Parirenyatwa hospital as well as published in a recognised journal. The scoping review findings will be used in a Theory of Change workshop together with information from focus group discussions and interviews to guide the formulation of a PC pathway for integrating into cervical and prostate cancer management at a tertiary hospital in Harare, Zimbabwe.

## Conclusion

This scoping review has identified PC models for cancer management at hospitals within SSA and showed the need for PC units/clinic/team at tertiary hospitals. The need for PC is increasingly acknowledged as a vital component of comprehensive cancer care in SSA. However, despite the expanding evidence base and policy interest, actual utilisation of PC services remains limited due to systemic, educational and infrastructural barriers. Gaps in PC utilisation were in awareness, integration models, clinician preparedness and health system support. The description of hospital ‘pathways’ for cancer patients who are admitted at hospitals, which was the stated objective had models, clinics, teams as the closest to describe what was intended since there were no studies identified using the term pathway. Another gap was the lack of description of the initiation and continuity of PC within the hospitals. Information obtained will contribute to the formulation of a PC pathway for cervical and prostate cancer at a tertiary hospital in Zimbabwe.

## Conflicts of interest

LF: The work reported herein was made possible through funding by the South African Medical Research Council through its Division of Research Capacity Development under the SAMRC Institutional Clinician Researcher Programme. The content hereof is the sole responsibility of the authors and does not necessarily represent the official views of the SAMRC. The rest of the authors have declared that no competing interest exists.

## Funding

Funder: National Institute for Health Care Research (NIHR) (GHRUG NIHR134440). Project Title: Global Health and Palliative Care (GHAP).

## Author contributions

ACT: Primary author. MB: Supervisor. ZMC: Supervisor. LF: Reviewer. NN: Supervisor. SR: Supervisor.

## Protocol registration

Open Science Framework (OSF): osf.io/rsd4q.

## Figures and Tables

**Table 1.** Summary findings from final included articles.

Authors	Title	Journal and Year Published	Country	Aim	Setting/ context	Study Population	Concept	Notes
Afessa *et al* [17]	PC service utilisation and associated factors among cancer patients at oncology units of public hospitals in Addis Ababa, Ethiopia	PLOS ONE 2024	Ethiopia	To assess PC service utilisation & associated factors affecting cancer patients at public hospitals oncology units in Addis Ababa, Ethiopia	Hospital: PC in oncology units	Adult cancer patients	Not described	PC service utilisation: predisposing, health need, enabling and health systems factors
Bonsu and Ncama [18]	Clinicians’ experiences and perspectives of breast cancer and possible integration of breast cancer prevention and early detection into PC.	International Journal of Africa Nursing Sciences 2019	Ghana	This study explores clinicians’ experiences and perspectives of breast cancer and possible the integration of breast cancer prevention and early detection into PC.	Teaching hospital: PC clinic in OPD	Clinicians	Model	Model of integration recommended. ‘Because our women come at advanced stage, chance of survival is very slim’, ‘All my patients do not survive the disease’. ‘There should be a protocol or a model that says that, do this and that, .in delivering care to the patients and families. This will guide any clinician who comes to work in the PC clinic. Hence, developing a model of care is paramount’
Ganca *et al* [19]	What are the communication skills and needs of doctors when communicating poor prognosis to patients and their families? A qualitative study from South Africa	South African Medical Journal 2016	South Africa	To explore communication skills and practices of medical practitioners when conveying a poor prognosis to patients and families, and to identify their communication skills, needs and understanding of PC	Tertiary hospital: PC team at hospital	Clinicians	Not mentioned	The emerging theory from this study is that doctors who understand the principles of PC and who have an established working relationship with a PC team feel supported and express low levels of emotional anxiety when conveying a poor prognosis. Having hospital-based PC teams in all public hospitals will provide support for patients and doctors handling difficult conversations.
Ogbenna *et al* [20]	Organisational models and patient-reported outcomes for PC across five tertiary hospitals in Nigeria: An Environmental Scan	PLOS Global Public Health, 2025	Nigeria	To reflect the quality of PC services through assessing patient-centered outcomes.	Tertiary hospitals: PC unit at each hospital	Leadership, frontline workers, patients and caregivers.	Model	The patients requiring PC are referred to the PC unit and vice versa.
Bonsu and Ncama [21]	Integration of breast cancer prevention and early detection into cancer PC model.	PLOS One 2019	Ghana	To develop a model to facilitate the integration of breast cancer prevention and early detection into cancer PC	Hospital PC clinic	Breast cancer patients, relatives, clinicians	Model	A proposed model of PC provision in the in the PC clinic at the hospital which starts from cancer screening and extends through the disease trajectory.
Cain and Denny [22]	PC in women’s cancer care: Global challenges and advances.	International Journal of Gynecology & Obstetrics 2018	South Africa	Not applicable	Not applicable	Not applicable	Model	Infrastructure to advance successful PC programs: Adequate human resources/training, Equipment/services, Policies and monitoring, Public awareness and education
Hamilton-Baillie *et al* [23]	PC in a rural sub-district in South Africa: A 4-year critical review.	African Journal of Primary Health Care & Family Medicine 2024	South Africa	To review the PC project over 4 years.	Rural clinic and hospital	All patients	Model	PC training has been prioritised. District-wide awareness around PC is actively maintained through regular training of staff, continuing medical education sessions and bedside and formal undergraduate and postgraduate student teaching.
Jacinto *et al* [24]	The prevalence of life-limiting illness at a Ugandan National Referral Hospital: a 1-day census of all admitted patients	BMJ Supportive & PC 2015	Uganda	To measure the proportion of all adult and child patients admitted with previously diagnosed active life-limiting disease, who therefore may be appropriate for PC provision, across all beds.	Hospital	All patients	Not mentioned	This study highlights the importance of hospital-based PC teams and, in light of data reporting the supply limitations of essential drugs in East African pharmacies, further study should develop and test hospital-based PC provision in SSA.
Kiyange *et al* [25]	Measuring PC integration in Malawi through service provision, access and training indicators: the Waterloo Coalition Initiative.	BMC PC 2024	Malawi	To measure PC integration based on 11 consensus-based indicators over a one-year period.	13 hospitals	Health professionals	Model	Demonstrating that increased PC access is possible in severely resource-constrained settings through sustained models of partnership at the local level.
LaVigne *et al* [26]	PC in Botswana: current state and challenges to further development.	Ann Palliat Med 2018	Botswana	To provide such a needs assessment, analysing not only the existing infrastructure for PC delivery, but also the ongoing challenges that PC development faces.	Hospital: PC confined to oncology ward	Nurses, physicians	Not mentioned	Infrastructural challenges such as access to pain medications, the strained size of the PC workforce, and a need for increased PC education and understanding.
Ndiok and Ncama [27]	Assessment of PC needs of patients/families living with cancer in a developing country.	Scand J Caring Sci 2018	Nigeria	To assess the care needs of oncology in-patients and clinic attendees or families in two tertiary health institutions.	Tertiary hospitals	Cancer patients, health workers	Not mentioned	Patients are able to deal with the disease more effectively when hospitals set up a PC team or unit to carry out proper assessment of patients living with cancer
Ndiok and Ncama [28]	Barriers and benefits of model development for integration of PC for cancer patients in a developing country: A qualitative study	International Journal of Nursing Science 2021	Nigeria	To identify barriers and benefits in establishing a model for integration of PC of cancer patients in daily clinical practice in tertiary health institutions.	Tertiary hospitals	Stakeholders. nurse managers	Benefits of the model were two-fold: hospital outcomes and patients/family outcomes.	Challenges to implementation of PC services in hospitals can be overcome by establishing workable policies and allocating adequate funds for PC activities. We purposively selected nurse managers because of their important role in patient care.
Tapera and Nyakabau [29]	Limited knowledge and access to PC among women with cervical cancer: an opportunity for integrating oncology and PC in Zimbabwe.	PLOS ONE Open access, 2020	Zimbabwe	To understand deeper issues, and to seek to explain surprising and unexpected results from the surveys.	Hospital	Patients	Not mentioned	PC which is predominantly provided by Island Hospice (an NGO) on referral basis.
Teklemariam *et al* [30]	Perception about PC and factors influencing the likelihood of PC service utilisation among adult cancer patients in Ethiopia	Journal of cancer care, WILEY 2022	Ethiopia	To assess the perception about PC and factors influencing the likelihood of PC service utilisation among adult cancer patients in Ethiopia	Tertiary referral hospital - cancer unit	Adult cancer patients	Not mentioned	10 adult cancer patients receive PC service from the cancer unit of the hospital daily (Dagne *et al*., 2019).
Ministry of Health and Child Care [31]	The National PC Policy	2014	Zimbabwe	1. Policy development 2. Health systems strengthening 3. capacity development	Not applicable	Patients, HCWs,	Models	Hospital Based PC Teams. The hospital has a team of professionals providing PC services in a hospital setting. The team receives patients from various departments and wards. PC is provided by the team within the hospital setting and in consultation with ward and departmental staff. Upon discharge from the hospital a follow up plan is made by the team together with a community nurse, the patient and family.

Key: OPD - Out patient’s department PC - Palliative care, SSA - sub-Saharan Africa.
